# Development and Analysis of a Three-Fin Trigate Q-FinFET for a 3 nm Technology Node with a Strained-Silicon Channel System

**DOI:** 10.3390/nano13101662

**Published:** 2023-05-18

**Authors:** Swagat Nanda, Rudra Sankar Dhar, Falah Awwad, Mousa I. Hussein

**Affiliations:** 1 Department of Electronics and Communication Engineering, National Institute of Technology Mizoram, Chaltlang, Aizawl 796012, Mizoram, India; nanda.swagat@gmail.com (S.N.); rudra.ece@nitmz.ac.in (R.S.D.); 2 Department of Electrical Engineering, United Arab Emirates University, Al Ain P.O. Box 15551, United Arab Emirates; f_awwad@uaeu.ac.ae

**Keywords:** TG Q-FinFET, three-fin structure, strained silicon, enhanced mobility, ballistic effect

## Abstract

Multi-gate field effect transistors (FETs) such as FinFETs are severely affected by short-channel effects (SCEs) below 14 nm technology nodes, with even taller fins incurring fringing capacitances. This leads to performance degradation of the devices, which inhibits further scaling of nanoFETs, deterring the progress of semiconductor industries. Therefore, research has not kept pace with the technological requirements of the International Roadmap for Devices and Systems (IRDS). Thus, the development of newer devices with superior performances in terms of higher ON currents, acceptable leakage currents and improved SCEs is needed to enable the continuance of integrated circuit (IC) technologies. The literature has advocated integration of strained-silicon technology in existing FinFETs, which is highly effective in enhancing ON currents through the strain effect. However, the ON currents can also be amplified by intensifying the number of fins in trigate (TG) FinFETs. Thus, three-fin TG quantum (Q)-FinFETs, using a novel tri-layered strained-silicon channel, are deployed here at 10 nm and 8 nm channel lengths. Threshold voltage is calculated analytically to validate the designs. The electrical parameters and quantum effects of both devices are explored, analysed and compared with respect to existing heterostructure-on-insulator (HOI) FinFETs and the proposed existing standard requirement of IRDS 2022 for a 3 nm technology node. The comparisons demonstrated a significant increase in the drive currents upon employing three fins of the same dimensions (8 nm gate length) and specifications in a device-based system. The performance is augmented in contrast to the 3 nm technology node device of IRDS 2022, with SCEs within the limits. Thus, employing a tri-layered strained-silicon channel system in each fin allowed for forming a three-fin Q-FinFET that, in our opinion, is the technique for consolidating the performance of the devices and enabling future generation device for faster switching operation in a sub-nano regime.

## 1. Introduction

With Moore’s prediction, the number of transistors that can be accommodated on an integrated circuit (IC) roughly doubles every 18 months, which leads to the shrinking of the transistor size and creates difficulties in fabricating them, due to their short-channel effects (SCEs) [1]. FinFET-based multi-gate (MuGFET) devices [2] were proposed long ago as a technology option for replacing the planar complementary metal oxide semiconductor (CMOS) to preserve the controllability of gate to channel for enriched performance with lessened leakages due to SCEs. Superior scalability, less leakage current, improved gate control and higher performance brand FinFETs more preferable than the existing linear technology devices. Tri-gate (TG) FinFET architecture has emerged, introduced by Kavalieros et al. [3] of Intel, being the device of choice at a gate length of 45 nm. This device utilised strain engineering to improve the ON current to 165 μA/μm, but the OFF current was determined to be quite high, at 139 nA/μm, with SCEs such as a subthreshold swing (SS) of 76 mV/decade and a drain-induced barrier lowering (DIBL) of 89 mV/V. FinFETs, being scaled further, have reached a limit for the drive currents, but the currents could be increased without affecting the unit footprint upon increasing the fin heights [4]. These taller fins are prone to increased fringing capacitance and series resistance, which hinders the electrical performance of the devices. Subsequently, an alternative approach emerged: increase the number of fins [5] in the same structure and achieve a trade-off in drive currents by balancing the fin heights and the number of fins. Hence, several types of FinFETs developed, divided according to their structure and usage, namely single-fin FinFETs, double-fin FinFETs, triple-fin FinFETs and quad-fin FinFETs. FinFETs are more preferred than the regular planar or bulk metal oxide semiconductor field effect transistors (MOSFETs) due to their enhanced scalability, better electrical controllability over the channel, and higher packing density [6], leading to multiple functions employed in the same space and lower power dissipation.

In general, nano devices are developed to have lower speeds with lower power consumption and higher speeds with higher power consumption, while the objective is to have higher speeds with lower power consumption [7]. Now, with multiple-fin systems created for FinFET technology, the device is equipped to achieve significantly higher speeds as well as relatively lower power consumption than the existing technologies. Henceforth, arrays of fins in FinFETs [8] are being implemented in today’s technological era, leading to higher speeds for device-based systems. However, with silicon or even with silicon-on-insulator (SOI)-array FinFETs [9] that are now being employed for various applications, there is a strenuous effort to be able to meet the need to be on par with the International Roadmap of Devices and Systems (IRDS) 2022 for the 3 nm technology node [10]. While Samsung and Intel have announced gate-all-around field effect transistor (GAA FET)-based devices such as MBCFET [11] and RibbonFET [12], respectively, they are still in the budding stages and are also costlier to design and fabricate in comparison to existing FinFETs. The 3D stacking, as a future IRDS proposal, is therefore not yet considered to have achieved the requirements. The researchers are thus facing great difficulties to meet the challenges, especially in designing and thereafter fabricating the same to match the requirements. To meet the purpose of acquiring faster device and RF applications, the existing and matured FinFET technology is employed here, since there is still more than enough room left to achieve the proposed performance of a 3 nm technology node as per IRDS 2022. Hence, the need stands to develop a multi-fin FinFET device system in a nano regime with extramural technological accomplishers to meet the requirements of enriched performance.

Strain engineering [13] is always an outstanding feature utilised for amplifying the drive currents in nanoFETs. Formerly, the literature classified strain techniques as global and local strain [14,15,16,17,18]. Strain incorporation for enhancement of the drive current in nFETs is achieved by channel engineering, though the literature also shows the incorporation of strain by doping the source/drain with SiGe to be an acceptable technique. Hoyt et al. [19] initially developed the strained-silicon techniques in FETs, inculcating strained-silicon (s-Si) on relaxed SiGe in the channel, and Kumar et al. [20] developed the dual strained layer channel (s-Si/s-SiGe) directly on the insulator. In the past two decades or so, development relating to performance enhancement has augmented electron mobility, transconductance and electron velocity, thereby enriching the drain current for textured nano devices that are spearheading the semiconductor markets. Recently, with hetero-structure-on-insulator (HOI) devices being developed by Khiangte et al. [21] for nanostructures, inducing the tri-layered strained-channel system is probably the future option that can be considered for enhanced performances. This device-based system, when indebted, leads to reduced threshold voltage (V_th_) due to the lowering of band gaps of Si with enhanced strain, incorporating carrier confinement and ballistic transport and instituting quantum-mechanical tunnelling through the ultra-thin channel of the device. Thereby, enrichment of mobility, drain current and transconductance by ~90%, ~33.3% and 11%, respectively, is observed [21] in comparison to the existing SOI devices. Thereafter, Kumar et al. [22] developed a strained quantum carrier confinement-based DG SHOI (strained heterostructure-on-insulator) FET with three ultra-thin layers within a channel region of 14 nm channel length, along with a gate underlap structure. With increase in underlap length, Kumar et al. [22] observed the drive currents to be reduced drastically. The 1 nm underlap device gracefully achieved ~25.3% reduction in DIBL in comparison to the 14 nm strained channel DG FET without underlap.

Bha et al. [23] designed FinFET devices at a nano regime with 10 nm gate length, having an SOI structure with a silicon channel, which achieved reduced leakage and high transconductance. Thereafter, Nanda et al. [24] simulated a quantum well barrier (QWB)- based TG FinFET, compiling the strain engineering with a channel length of 10 nm. The device presented improved control over the SCEs and an enhancement of 44% in the drain current when contrasted with the FinFET designed by Bha et al. [23] of the same channel length. Even though the use of strained-silicon channels enhances the drive currents, considering the requirement of applications with higher speeds and lower energy consumption, there is still a need to increase the current further by increasing the number of fins; developing a multi-fin FinFET device system is the consensus.

With the above concepts already applied in today’s technology, the motivation of this paper remains to develop a multi-fin (three-fin) FinFET structure with a hetero-strained channel system that can pursue the fundamental desire for devices with higher speeds and lower power consumption while being on par with the proposed IRDS 2022 requirements for a 3 nm technology node. Hence, the core focus remains to develop and design three-fin Quantum (Q) FinFETs with 10 nm and 8 nm gate lengths, inducing a three-layered strained nano-channel system by incubating a quantum barrier consisting of s-Si/s-SiGe/s-Si in the device. The devices developed here are analysed, characterised and explored for their electrical parameters based on fundamental analytical calculations and compared to that of a single-fin TG n-channel FinFET of 10 nm channel length. The device is optimised for enhanced observations, possibly leading to a healthier and improved device that satisfies needs while being on par with technological advancements.

## 2. Device Structure and Theory

**Figure 1:**
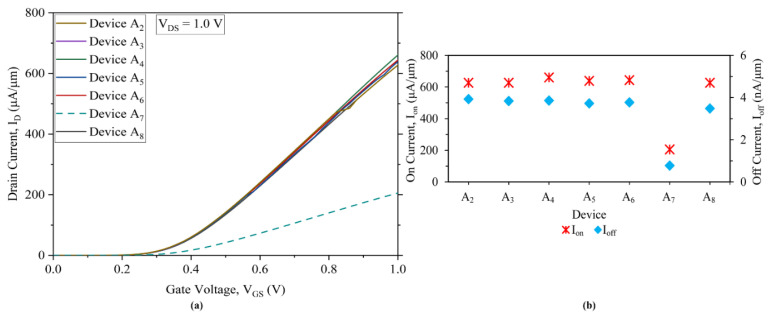
(**a**) Linear scale I_D_–V_GS_ transfer characteristics for three-fin devices A_2_–A_8_ at V_DS_ = 1 V. (**b**) Comparison of ON and OFF currents of three-fin devices A_2_–A_8_ at V_DS_ = 1 V.

Considering the core objective of developing a device to meet the requirement of higher speed and thereby, a faster device, the HOI nanostructured system device consisting of three fins is employed here for the first time. Initially, a 10 nm gate-length-based three-fin device is developed on a Si substrate and a buried oxide (BOX) layer, attaining a combined height of ~80 nm; this device is designated as A_1_ and listed in Table 1. The gate terminal of this device is formed by depositing a 1 nm layer of SiO_2_ on three sides of the hetero-channel, above which a 2 nm layer of conductor is settled. The fins for the devices are separated by a gap of 4 nm. This gap consists of 1 nm of SiO_2_ on each side of two fins, with a 2 nm layer of conductor in between. When the gate length is scaled down to 8 nm, the total device features a very large size in contrast to the active region (channel) of the device. Therefore, to optimise the area of the active region, a varied number of nano devices, labelled A_2_–A_8_, are developed. In these devices, the BOX-and-substrate heights are gradually decreased by steps of 10 nm to determine the effect of the buried oxide layer on electric field penetration in the substrate of the device. Devices A_2_–A_6_ are designed with a decreasing BOX-and-substrate height from 80 nm to 40 nm while maintaining a gap of 4 nm between the fins, as developed for Device A_1_. However, when the BOX-and-substrate height reached 30 nm, two more devices were developed. One of the devices, which is labelled A_7_, is designed with fins separated by a gap of 6 nm. This consists of 1 nm of SiO_2_ on each side of two fins and a 4 nm layer of conductor in between. Another device, labelled A_8_, has the same gap of 4 nm between the fins as in A_2_–A_6_. These devices, labelled A_2_–A_8,_ are tabulated in Table 1. The gradual decrease in the thickness of BOX-and-substrate and its effect on the electrical characteristics are consistently observed to have negligible impact on the device characteristics. The linear transfer characteristics of devices A_2_–A_8_ are plotted in Figure 1a. It can already be seen that there is only a trivial change in the drive currents, leakage currents and the I_on_/I_off_ current ratio due to full dispersion of the electric field within the BOX without affecting the substrate, as revealed in Figure 1b and listed in Table 2. For the 8 nm gate length device, A_7_, the gate metal is 4 nm thick between the two fins and therefore exhibits smaller leakage currents and a higher I_on_/I_off_ current ratio in comparison to the other 8 nm devices. However, the ON current is not adequate for performance consideration due to increased fringing capacitances that interact with each other as a parasitic effect and in turn degrade the drive current; hence, Device A_7_ is discarded. With a compact device and space utilisation being the requirement and observing nearly no interacting fringe-fin effects, the novel 8 nm three-fin device is further evolved with 2 nm gate metal thickness separating the two fins; it is labelled A_8,_ as in Table 1. This device has an ON current and leakage current on par with devices A_2_–A_6_, but with a smaller die area; therefore, it is the device under consideration. The penetrating electric field is also observed to be negligible and under control, up to a 30 nm combined height of BOX-and-substrate (20 nm and 10 nm). Thinning the BOX-and-substrate below 30 nm eliminates effectiveness in holding the device active region on top; hence, this option is not considered.

**Figure 2:**
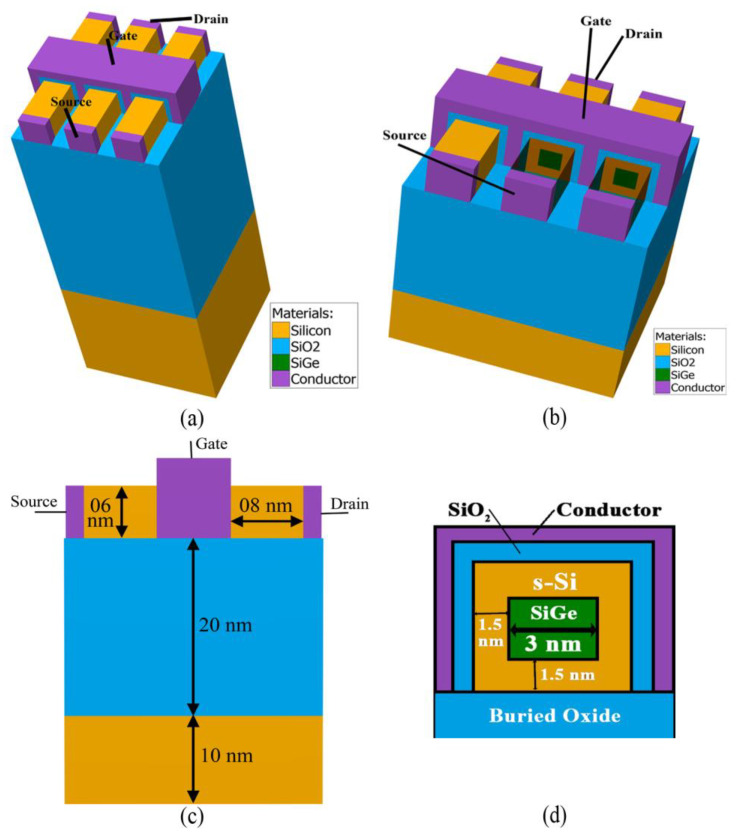
3D representation of the developed TG strained-channel three-fin Q-FinFET (**a**) 10 nm gate length; (**b**) 8 nm gate length; (**c**) dimensions of different regions of the 8 nm Q-FinFET; (**d**) cross-sectional view of the tri-layered channel of each fin, detailing the dimensions.

The novel tri-layered structure for each fin of the FinFET in devices A_2_–A_8_ comprises a s-Si/s-SiGe/s-Si-based ultrathin nano-channel system with gate length of 8 nm and a single metal gate conductor. For 10 nm three-fin Device A_1_, the combined height of BOX-atop-substrate is finalised as ~80 nm, with the individual fins separated by 4 nm, whose 3D structure is shown in Figure 2a. The top views of the full structures of devices A_1_ and A_8_ are presented in Figure 2a and Figure 2b, respectively, where the length of the source and drain of each of the fins of A_1_ and A_8_ devices are fixed at 10 nm and 8 nm, respectively. Considering the concept of keeping the die area smaller, as mentioned earlier, a substrate of 10 nm followed by a 20 nm layer of BOX is grown to form the finalised base region onto which the novel quantum three-fin FinFET (A_8_) with n-channel at 8 nm gate length is developed, as in Figure 2c. The individual heights and widths of the fins of both devices are finalised to be 6 nm, with a combination of 1.5 nm/3 nm/1.5 nm (s-Si/s-SiGe/s-Si), which is favourably seen from the cross-sectional view in Figure 2d. Earlier research proposed a technique to improve drive currents with tall FinFETs on bulk Si and a high aspect ratio, as these taller fin devices indebt enhanced current performance. However, these are prone to increasing fringing capacitance and series resistance, which hinders the electrical performances of the devices. Saha et al. [25] investigated the effects of different aspect ratios on electrical characteristics and concluded that square devices having equal heights and widths, with an aspect ratio of unity, have superior results. Table 3 therefore presents the optimised novel 10 nm three-fin Q-FinFET as Device A_1_ and the novel 8 nm optimised three-fin Q-FinFET as device A_8_, while the existing 10 nm HOI TG FinFET of Nanda et al. [24] for calibration and validation is termed Device A_9_.

The doping concentration of the source is 5 × 10^18^ cm^−3^ and that of drain is 1 × 10^18^ cm^−3^ with n-type silicon, while the channel and substrate are doped with doping concentration of 1 × 10^15^ cm^−3^ p-type silicon. The difference in doping of the source and drain assures the negligible effects of hot carriers, especially in the channel region of the novel nano devices. The detailed parameters considered for the novel three-fin Q-FinFET are laid out in Table 3.

The channels in each of the fins for Devices A_1_ to A_8_ are developed as per the tri-layered hetero-strained nano-channel system. Thus, double s-Si layers are achieved in each of the fins, which forms the Type-II band system within the ultrathin layers, instigating quantum carrier confinement at the interfaces and inducing ballistic transport to enhance the mobility of the charge carriers in the devices; this leads to a significant increase in the drive currents. Considering the confinement effect and quantisation theorem and subsequent estimation of the fundamentals of the TG FinFET, the threshold voltage of the TG silicon-on-insulator (SOI) FinFET due to strained-silicon channel needs to be formulated so as to analytically validate the novel device developed here. This is regulated on the basis of 2D analysis of the inversion carrier sheet density Q_inv_DGF_ at the virtual cathode position of a double-gate FET (DGFET) [26], which is determined as follows:

(1) $$Q_{inv\_DGF}=n_{i}W_{Fin}\left\{exp\left(\frac{{∆\phi }_{m}\_DG}{V_{th}}\right)\right\}exp\left(\frac{V_{g}-V_{flatband,Si}}{V_{th}}\right)$$

where n_i_ is the intrinsic carrier concentration; W_Fin_ is the width of the device; ${∆\phi }_{m}\_DG$ is the extra potential in the DGFET device, which is dependent on both the drain and gate potential; V_g_ is the gate voltage; V_flatband,Si_ is the flat-band voltage of the silicon channel; and V_th_ = kT/q.

The threshold voltage of a TG FinFET is therefore derived from the DGFET by considering the effect of the top gate as one symmetric and one asymmetric DGFET. The effect of the asymmetric DGFET is neglected in this case, as the TG FinFET devices employed and developed here have equal height and width. For the symmetric DGFET, the surface potential is derived upon solving the Poisson equation and is of the type

(2) $$\phi \left(x,y\right)=a_{1}\left(y\right)+a_{2}\left(y\right)x+a_{3}\left(y\right)x^{2}$$

where a_1_(y), a_2_(y), a_3_(y) are functions of fin height only, as considered by Panchanan et al. [26].

Now, upon inserting the boundary conditions ϕ_s_ = V_bi_ at the source end and ϕ_s_ = V_bi_ + V_d_ at the drain end, where V_bi_ is the built-in potential and V_d_ is the drain potential, the surface potential is determined. In the channel of the DGFET, the extra potential Δϕ_m_DG_ is thus represented by

(3) $${∆\phi }_{m\_DG}=\phi \left(x,y\right)-A_{sym}$$

where

(4) $$A_{sym}=-\left\{V_{g}^{\prime }-\left\{\left(\frac{{qN}_{A}}{2\varepsilon_{ox}\varepsilon_{Si}}\right)\left\{\varepsilon_{Si}t_{ox}W_{Fin}+\varepsilon_{ox}\left(W_{Fin}-x\right)x\right\}\right\}\right\}$$

and $V_{g}^{\prime }=V_{g}-V_{flatband,Si}$, N_A_ being the doping concentration in the channel, $\varepsilon_{Si}$ being the permittivity of silicon, $\varepsilon_{ox}$ and $t_{ox}$ being the permittivity and thickness of the SiO_2_ gate oxide layer, respectively, and q being the electronic charge.

Thus, for the symmetric DGFET, we calculate the threshold voltage $V_{T,DGsym}$ as

(5) $$V_{T,DGsym}=V_{flatband,Si}-\frac{C_{1sym}\left(V_{bi}+V_{d}\right)+C_{2sym}V_{bi}}{1-\left(C_{1sym}+C_{2sym}\right)}+\frac{V_{th}}{1-\left(C_{1sym}+C_{2sym}\right)}ln\left(\frac{Q_{inv\_DGF}}{n_{i}W_{Fin}}\right)$$

where $C_{1sym}$ and $C_{2sym}$ are constants that are exponential functions of the channel length and the virtual cathode position where the potential is lowest, as conceptualised by Panchanan et al. [26].

The inversion charge of a TG FinFET, Q_th_TGF_, is expressed as a scaled factor of the inversion charge density of DGFET, and is written as

(6) $$Q_{th\_TGF}=\alpha \cdot H_{Fin}\cdot Q_{inv\_DGF}$$

where H_Fin_ is the fin height, and α is the scaling factor, which is always greater than unity, considering the effect of the top-gate oxide of the TG FinFET. Thus, for the TG FinFET, the inversion or threshold charge, Q_th_TGF_, becomes

(7) $$Q_{th\_TGF}={\alpha n}_{i}W_{Fin}H_{Fin}\left\{exp\left(\frac{{∆\phi }_{m}\_DG}{V_{th}}\right)\right\}exp\left(\frac{V_{g}-V_{flatband,Si}}{V_{th}}\right)$$

Now, by using similar techniques, the threshold voltage of the TG FinFET, $V_{T,TGF}$, is determinedc combining Equations (5) and (7) as

(8) $$V_{T,TGF}=V_{flatband,Si}-\frac{\frac{2H_{Fin}C_{1sym}}{W_{Fin}+{2H}_{Fin}}\left(V_{bi}+V_{d}\right)+\frac{2H_{Fin}C_{2sym}}{W_{Fin}+{2H}_{Fin}}V_{bi}}{1-\left(C_{1,TG}+C_{2,TG}\right)}+\frac{V_{th}}{1-\left(C_{1,TG}+C_{2,TG}\right)}ln\left(\frac{Q_{th\_TGF}}{{\alpha n}_{i}W_{Fin}H_{Fin}}\right)$$

In this present device, since W_Fin_ = H_Fin_ dimensionally, Equation (8) reduces to

(9) $$V_{T,TGF}=V_{flatband,Si}-\frac{\frac{2}{3}C_{1sym}\left(V_{bi}+V_{d}\right)+\frac{2}{3}{C_{2sym}V}_{bi}}{1-\left(C_{1,TG}+C_{2,TG}\right)}+\frac{V_{th}}{1-\left(C_{1,TG}+C_{2,TG}\right)}ln\left(\frac{Q_{th\_TGF}}{{\alpha n}_{i}W_{Fin}H_{Fin}}\right)$$

Since the channel region consists of two strained-silicon regions with thickness of d_s-Si_ and one strained SiGe region of thickness of d_s-SiGe_, the strain generated in the channel is λ_strain,_ and the fin width [27] is calculated to be

(10) $$\lambda_{strain}=2\left(\frac{d_{s-SiGe}-d_{s-Si}}{d_{s-SiGe}}\right)\;\&\;W_{Fin}=(2d_{s-Si}+d_{s-SiGe})$$

Replacing W_Fin_ from Equation (10) in Equation (9), the threshold voltage of the HOI FinFET is developed as

(11) $$V_{T,TGF}=V_{flatband,s-Si}-\frac{\frac{2}{3}C_{1sym}\left(V_{bi}+V_{d}\right)+\frac{2}{3}{C_{2sym}V}_{bi}}{1-\left(C_{1,TG}+C_{2,TG}\right)}+\frac{V_{th}}{1-\left(C_{1,TG}+C_{2,TG}\right)}ln\left(\frac{Q_{th\_TGF}}{{\alpha n}_{i}H_{Fin}\left(2d_{s-Si}+d_{s-SiGe}\right)}\right)$$

where V_flatband,s-Si_ represents the flatband voltage of the tri-layered strained-silicon channel.

Due to the presence of the strained-silicon channel, the flatband voltage is also modified [28], because of the band bending and splitting effect, to be

(12) $$V_{flatband,s-Si}=V_{flatband,Si}+{∆V}_{flatband}$$

where

(13) $$V_{flatband,Si}=\emptyset_{M}-\emptyset_{Si}$$

(14) $${∆V}_{flatband}=\frac{-{\left({∆E}_{c}\right)}_{s-Si}}{q}+\frac{-{\left({∆E}_{g}\right)}_{s-Si}}{q}-V_{th}ln\left(\frac{N_{V,Si}}{N_{V,s-Si}}\right)$$

where $\emptyset_{M}$ is the gate work function; $\emptyset_{Si}$ is the silicon work function without strain; ${\left({∆E}_{c}\right)}_{s-Si}$ is the electron affinity change in silicon due to strain; ${\left({∆E}_{g}\right)}_{s-Si}$ is the bandgap decrease in silicon due to strain; $N_{V,Si}$ and $N_{V,s-Si}$ are the density of states in unstrained and strained silicon, respectively; and V_th_ = kT/q, with q being the electronic charge.

Based on this calculation, the threshold voltage of the strain-induced quantum three-fin FinFET devices is determined, and the transfer characteristics are extracted. The related parametric analysis of the strained nano device is further investigated and carried out using Silvaco tools [29], assimilating various models such as the band gap narrowing model, the Lombardi CVT model, the SRH model, the Auger model and the Arora model, along with Hansch quantum models for analysing the performance that are based on device parameters such as current density effect, electron mobility, electric field, electrostatic potential, charge density energy band gap and electron velocity. The Hansch quantum model is included to provide a precise estimate of the quantum confinement effects in the inversion layers of the well-barrier channel in ultra-thin HOI devices having three-fin structures. In this ultra-thin strained channel nano device, the model accounts for the carrier confinement effects in the inversion layers and presents a quantitative estimation of the probable enrichment in mobility, electrostatic potential and electron velocity in the device, which is also compared and calibrated with existing devices for enrichment analysis. The ON current and, finally, the current density across the channel regions are thereafter assessed and a qualitative analysis is overlaid for the novel strained tri-layered three-fin Q-FinFET device.

## 3. Results and Discussion

### 3.1. Electrical Characteristics of Three-Fin Quantum FinFETs

**Figure 3:**
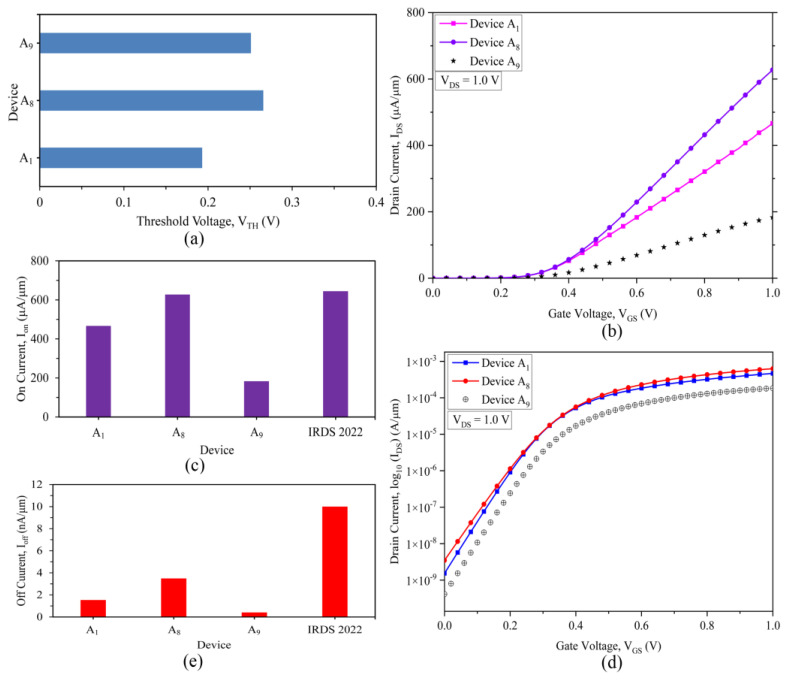
(**a**) Threshold voltages of the three-fin 10 nm (Device A_1_) and 8 nm (Device A_8_) gate length FinFETs compared with Device A_9_; (**b**) Linear scale I_D_–V_GS_ transfer characteristics for three-fin Devices A_1_ and A_8_ at V_DS_ = 1 V, (**c**) ON Current (I_on_) performance of the three-fin 10 nm (Device A_1_) and 8 nm (Device A_8_) gate length Q-FinFETs compared with Device A_9_ and IRDS 2022, (**d**) Logarithmic scale I_D_–V_GS_ characteristics of three-fin quantum FinFETs (Devices A_1_ and A_8_) at V_DS_ = 1 V, (**e**) OFF Current (I_off_) analysis of the three-fin 10 nm and 8 nm (Devices A_1_ and A_8_) gate length Q-FinFETs compared with Device A_9_ and IRDS 2022.

The strained channel novel three-fin FinFETs with 8 nm and 10 nm gate length are developed here for the first time, with tri-layered nano-channel (s-Si/s-SiGe/s-Si)-based fins in the system having thicknesses as described in Table 3. The calculated threshold voltage, V_th_, from Equation (11), for the 10 nm and 8 nm strained channel three-fin nano devices (Device A_1_ and Device A_8_) are acquired, presented and compared with the existing 10 nm single-fin FinFET (Device A_9_) of Nanda et al. [24] in Figure 3a. It is observed that the threshold voltage of A_9_ is 0.251 V [24], while the calculated threshold voltages of A_1_ and A_8_ novel three-fin devices are 0.193 V and 0.265 V, respectively, which is close to the single-fin device. The threshold voltages of the three-fin Q-FinFETs are determined from the simulations to be 0.199 V and 0.268 V for Device A_1_ and A_8_, respectively, which is analogous to the calculated values, thereby validating the simulated novel A_1_ and A_8_ devices. In order to minimise the impacts of short-channel effects and maintain an optimised threshold voltage for probable enriched performances, the overall channel thickness is kept closer to the length of the gate (10 nm/8 nm). Although the increase in number of fins affects the drain currents, the threshold voltage seems to be unaffected, resulting in the likely improvement in leakage currents. The threshold voltage of the device being equivalent to that of a single-fin device, the novel devices assist in excellent but faster switching control, providing an overall excellent effect of the device.

The linear transfer characteristics of the three-fin FinFET devices are acquired on the basis of the threshold voltage of the devices. The I_D_–V_GS_ plots for the 8 nm gate length Devices A_2_–A_8_ showed the same overlapping results; therefore, A_8_ is presented and compared with the 10 nm gate length Device A_1_ in Figure 3b. The maximum ON current, I_on_, is extracted from the linear plot of the I_D_–V_GS_ characteristics for further comparison and analysis for the Q-FinFETs. I_on_ refers to the drive current, or the ON current, when the gate voltage reaches the supply voltage. The I_on_ current witnessed for the three-fin HOI devices at a drain voltage of 1.0 V is determined to be 466.63 µA/µm for Device A_1_, and 627.25 µA/µm for Device A_8_. The I_on_ of devices A_1_ and A_8_ represents a ~154% and ~242% enhancement over Device A_9_, respectively, as revealed by Figure 3c, which demonstrates the impact of developing a three-fin technology device and implementing it to provide enhanced performance, while meeting the requirements as desired by IRDS 2022 for a 3 nm technology node [10]. Since each of the fins in the three-fin quantum devices employs ultra-thin s-Si regions, the charge carriers are confined to these regions, providing a cumulative quantised effect while travelling across the channel region with minimal scattering, leading to quasi-ballistic transport. This phenomenon, as inducted with multi-fin architecture, results in a tremendous increase in the ON currents of the devices. The specifications of IRDS 2022 for a 3 nm technology node for the ON currents of HD devices (not designed for) is achieved by Device A_8_, presenting itself as the superior device while suggesting the 8 nm three-fin Q-FinFET as the probable device of choice for the future generation.

The I_D_–V_GS_ characteristics in the logarithmic scale, as observed in Figure 3d, determine the leakage current and account for other short-channel effects such as subthreshold swing and DIBL of the novel quantum devices. The leakage current is determined to be current across the device when the gate and drain voltages are both equal to 0, ideally. With an increased number of fins, the leakage currents are bound to upsurge due to the combined effect of the individual fins. As detected in Figure 3e, the leakage currents of Devices A_1_ and A_8_ are determined to be 1.54, and 3.49 nA/µm, respectively, which is also an improvement in comparison to the standards suggested by IRDS 2022 [10] for a 3 nm technology node device; thereby the novel Device A_8_ profoundly supersedes present devices while providing an augmented feature relative to those desired for a 3 nm technology node, even with a device of 8 nm gate length.

**Figure 4:**
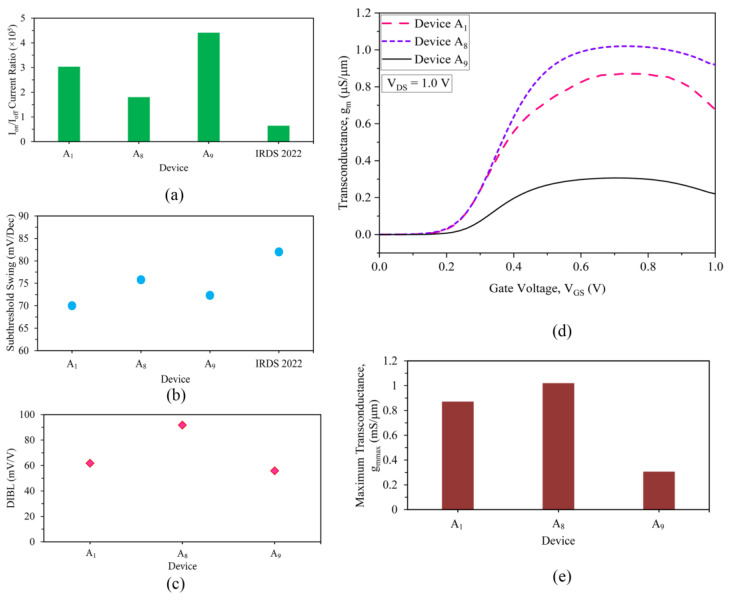
The three-fin quantum FinFETs of 10 nm and 8 nm (Device A_1_ and A_8_) gate length contrasted with Device A_9_ and IRDS 2022 for (**a**) I_on_/I_off_ current ratio and (**b**) subthreshold swing (SS). Comparison analysis of (**c**) calculated DIBL values, (**d**) transconductance (g_m_) vs. V_GS_ plot, and (**e**) maximum transconductance (g_mmax_) calculation plots of the three-fin 10 nm (Device A_1_) and 8 nm (Device A_8_) gate length FinFETs compared with existing Device A_9_.

The I_on_/I_off_ current ratios are thereafter calculated to be 3.03 × 10^5^ and 1.8 × 10^5^ for Devices A_1_ and A_8_, respectively, and presented in Figure 4a; they are almost an order of magnitude higher than the calculated values extracted from the 3 nm technology node device postulations of IRDS 2022. Hence, the 8 nm gate length three-fin strained channel HOI FinFET stands as the most suitable device of the future. The subthreshold swing (SS), as mentioned in IRDS 2022 for HP Devices, is 82 mV/Decade, but the novel strained quantum devices are observed to be far better. The SS is observed to be around 71 and 76 mV/decade for the three-fin Devices A_1_ and A_8_, demonstrating superior performance. The SS of these devices is also compared with Device A_9_ (73 mV/Decade) and are found to be on par with the single-fin HOI device, as evidenced in Figure 4b.

The drain-induced barrier lowering (DIBL) of A_1_ and A_8_ is deduced to be 61.8 mV/V and 91.78 mV/V, respectively as displayed in Figure 4c; these are comparable to the single-fin HOI FinFET device, thereby showing fabulous control of SCEs in these three-fin quantum devices. The three-fin 10 nm nano Device A_1_ shows only a ~10.7% increase in the DIBL, as compared to a 154% enhancement in drive currents.

The RF performances of the novel devices are characterised by analysing the transconductance (g_m_) of the devices, and Figure 4d plots the g_m_ of the novel 10 nm and 8 nm three-fin nanoFET devices A_1_ and A_8_ and compares it with the existing 10 nm HOI single-fin FinFET Device A_9_. At V_DS_ = 1.0 V, the extraction of the maximum transconductance g_mmax_ yields a value of 0.871 and 1.020 mS/μm for devices A_1_ and A_8_, respectively, which is a massive 184.6% and 233.3% enhancement in comparison to the 10 nm HOI FinFET Device A_9_, as apparent in Figure 4e. A higher transconductance value enriches the gain of the device, thereby making it appropriate for RF applications; this is obtained for both Device A_1_ and A_8_.

The comparison of electrical characteristics of the 10 nm and 8 nm channel length devices A_1_ (this study) and A8 (this study) is accomplished with the FinFET established by Kavalieros et al. [3] of Intel using strain engineering, the 10 nm DG SHOI FET developed by Kumar et al. [22], the 10 nm SOI FinFET of Bha et al. [23], and the 10 nm QWB FinFET piloted by Nanda et al. [24]. These comparisons are presented in Table 4. The ON current of A8 is determined to be far superior to all other devices along with minimal variations in leakage currents and other SCEs, thereby determining A_8_ to be the device of the future.

### 3.2. Investigation of Quantum Effects in Three-Fin FinFETs

**Figure 5:**
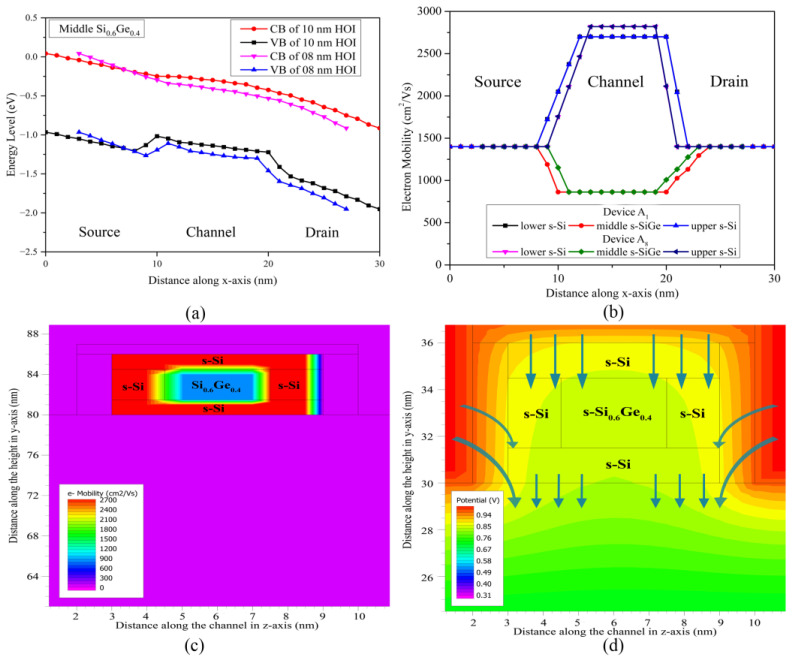
(**a**) Energy band diagram along the length (through the s-SiGe region of channel) of a fin, comparing 10 nm and 8 nm three-fin Q-FinFETs; (**b**) Average electron mobility along the length of individual fins of the strained-channel 10 nm and 8 nm three-fin Q-FinFETs; (**c**) Electron mobility contours across the channel of one fin of the 10 nm three-fin Q-FinFET; (**d**) Potential variation across the width of the channel of a single fin of Device A_8_.

On analysing the electrical characteristics of the devices, the quantum effects in the nano device in terms of electron mobility, electron velocity, current density and other associated parameters needs to be investigated. As the device dimensions are scaled below 10 nm gate length, the quantum effects are obvious and can no longer be neglected, as they play a significant role in the flow of charge carriers across the channel. In order to incorporate the effects of quantum barriers on the devices, the Hansch quantum model is included in the simulation of the devices. Since the channel consists of an ultra-thin s-SiGe region surrounded by two s-Si regions on all sides, it leads to the formation of a Type-II square finite quantum well-barrier system in the nano-channel of the novel three-fin strained HOI FinFETs. Since the electrical characteristics are considered at a drain voltage of 1.0 V, these quantum effects are also studied at V_GS_ = V_DS_ = 1.0 V. The energy band diagrams of the 10 nm and 8 nm three-fin FinFETs are plotted in Figure 5a. The conduction band shows a gradual decrease from the source to the drain through the s-SiGe region of the channel due to the application of high drain voltage. However, the valence band of both the devices shows a significant rise in energy levels inside the middle s-SiGe region, leading to the formation of a finite quantum barrier in the nano device. The quantum barrier formed by Device A_8_ is slightly lower in potential than the barrier formed by Device A_1_, due to reduced overall dimensions of A_8_. This leads to the blockage of charge carriers through the s-SiGe layers of all the fins of A_1_ and A_8_, resulting in current flow only through the s-Si layers. As the electrons transport through the s-Si regions, their mobility increases as a consequence of lattice mismatch between the silicon and SiGe layers, thereby inducing band bending via the splitting of energy levels in the form of four-fold and two-fold valleys [30]. The electron mobility along the length of the device is derived in Figure 5b. The electron mobility displays a significant rise in the s-Si regions of the channel, as the quantum barriers does not allow the electrons to pass through the middle s-SiGe regions, where quantum carrier confinement is indebted.

The electron mobility contours along the entire width of the 8 nm three-fin device exhibit identical mobility variations across all the three fins of the device. Therefore, it is certain that all the three fins in the nano device behave and perform equally; cumulatively, they provide for the total performance of the device. Henceforth, these variations are observed and presented only across a single fin for the novel three-fin FinFET device. The electron mobility contours along the cross-section of the channel are portrayed in Figure 5c, and it is observed that the mobility is highest in the ultra-thin s-Si regions that gradually decrease towards the middle s-SiGe region due to the finite quantum barrier formed in the channel, attributed to the Type-II hetero-band system deployed by the lattice mismatch of the s-Si and s-SiGe layers, as clearly detected in Figure 5a, resulting in quasi-ballistic transport of charge carriers. Due to the short channel length (8 nm and 10 nm) for all devices (A_1_ to A_8_) developed here, ballistic transport overrules and negligible roughness scattering inducts, as is clearly observed from the mobility graphs (Figure 5b,c) showing little variation in the channel for the transport of electrons from source to drain through the strained-Si layer. At the same time, a slight reduction in carrier mobility is observed in line with the findings of Mizuno et al. [31], due to the influence of negligible roughness at the strained-Si–SiGe interface. Thus, the electrons are accelerated with a negligible scattering effect in the s-Si channel regions, achieving velocities higher than the electron saturation velocities; in turn, this induces a phenomenon leading to the succumbing of the charge carrier in the channel regions of the nano device. Additionally, it is to be noticed from Figure 5c that at the Si/SiGe interfaces throughout the channel region, the mobility is observed to decrease intermittently as quantum tunnelling-based electron-hole recombination of carriers occurs; this forms a dipole, employing a pinning effect across the interface regions due to the hole carrier being confined in the s-SiGe barrier regions, which interact with neighbouring electrons transporting through the s-Si layers. However, this affects the device performance negligibly. Thereby, the ballistic transport of electrons occurring without a scattering effect in the nano-channel of the quantum FinFET device suffices for its superior performance.

**Figure 6:**
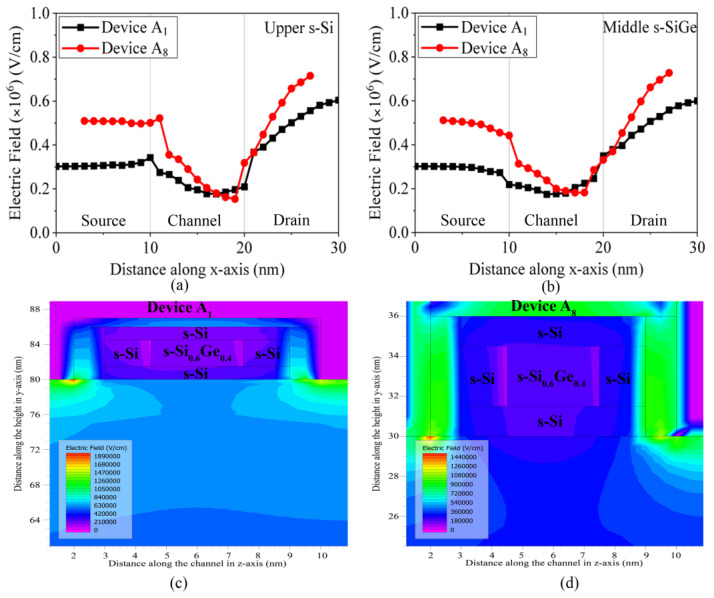
Electric field variations along the length of Device A_1_ and A_8,_ across the length of the channel (**a**) upper s-Si region, (**b**) middle s-SiGe. Contours of electric field for the FinFET devices: (**c**) 10 nm three-fin Q-FinFET and (**d**) 8 nm three-fin Q-FinFET.

The electric field across the length of the channel is plotted in Figure 6a for devices A_1_ and A_8_ across the upper s-Si channel and in Figure 6b across the middle s-SiGe layer of the channel. Since the lower s-Si is not under the influence of either gate or drain voltage, both the transverse and lateral electric fields deliver negligible impacts on the total performance, hence their effect is not considered for the observations. The graphs in Figure 6a,b convey that the electric field is lower in the source in comparison to the drain, which is attributed to the higher doping profile of the source. However, the electric field at the source-to-channel interface is higher than the electric field of the drain-to-channel interface. This is a consequence of the lower electrical conductivity in the channel layers due to lower p-type doping levels, attributed to the decrease in the electric field across this region. The contour diagrams of Figure 6c,d of devices A_1_ and A_8_ clearly illustrate that the electric field is spread only across the gate and its dielectric regions of the devices and does not penetrate into the channel regions, thereby demonstrating the fact that SiO_2_ (gate oxide) serves its purpose by sufficiently blocking the electric field in the channel region of the device. In contrast, as the gate length is reduced to 8 nm for Device A_8_, the effectiveness of the gate oxide seems to be not as beneficial as in A_1_, with a high field effect enticing the oxide, leading to minimal corner effects that sabotage part of the s-Si region in the channel. Thus, it can be interpreted that although 1 nm SiO_2_ is sufficient to block the electric field in A_1_, the same is not equally true for Device A_8_, as leakage is detected in Figure 3d, though it is well within the limits as per the 3 nm technology node of IRDS 2022. Thus, the 1 nm insulator does not degrade the performance for the novel three-fin Q-FinFET structure.

**Figure 7:**
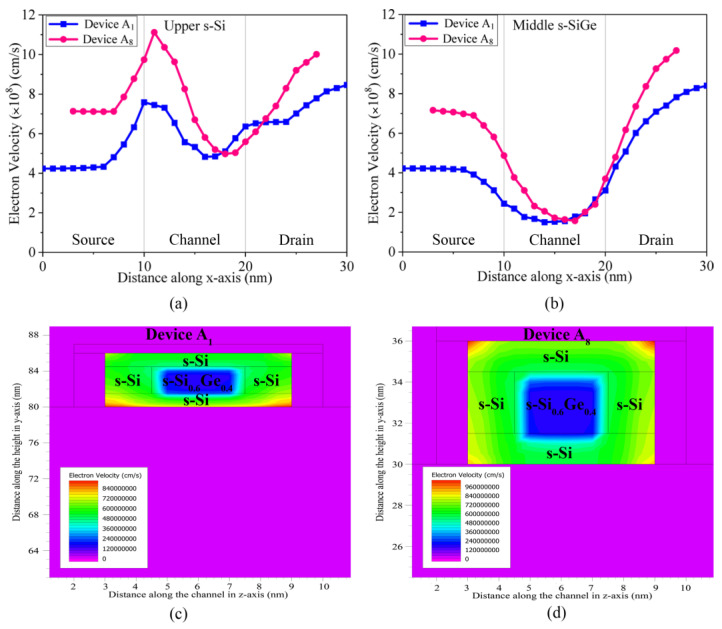
Electron velocity variations along the length of Device A_1_ and A_8_ across the channel (**a**) upper s-Si region (**b**) middle s-SiGe. Contours of electron velocity across the channel for the devices (**c**) 10 nm three-fin Q-FinFET and (**d**) 8 nm three-fin Q-FinFET.

The electron velocities of the devices are analysed and presented in Figure 7. It is already established that the formation of a finite quantum barrier due to the s-SiGe layer in the channel of each fin of the quantum devices restricts the flow of electrons only to the s-Si regions. As the electrons are accelerated towards the ultra-thin s-Si regions from the source, they easily transport through because of the loosened atomic structure, attributed to the lattice mismatch of the Si and SiGe regions, which institutes a band-bending effect in the channel. This leads to increased velocities of the charge carriers, often increasing beyond their saturation velocities, and leads to ballistic transport with minimal phonon scattering; this results in a significant increase in the drain currents of the device. Thus, with three fins in the novel devices A_1_ and A_8_, an increase in the drain current occurs as a cumulative effect of the performances. In Figure 7a,b, it is noticeable that the electron velocities of the 8 nm device are higher than of the 10 nm device due to shrunk device dimensions. Moreover, the electron velocity in the drain is always higher than the source. This is attributed to lower doping as well as a high positive charge in the drain, which allows for faster transport of the charge carriers from the drain-to-channel interface to the drain terminal, thereby inducing the phenomenon of succumbing of charge carriers across the channel of the quantum device. In Figure 7a, the electron velocity increases at the source-to-s-Si interface, while it shows a significant dip in Figure 7b at the source-to-s-SiGe interface, caused by the formation of a quantum well-barrier. A dipole is thereby created, with the confined hole carriers of the SiGe layer and the transporting electrons of the s-Si layers conserving a pinning effect within the channel region. This effect is further observed in Figure 7c,d, with the green contours indicating the variations in electron velocity, with higher velocities penetrating through the s-Si/s-SiGe interface barriers across the width of the channel. The velocity in the s-Si regions reaches a value of ~6 × 10^8^ cm/s, which is much higher than the saturation velocities, while the dip in the s-SiGe region is inevitable. This confirms that the ballistic transport of the charge carriers occurs in these s-Si regions without phonon scattering, resulting in drastic enhancement of the drain current. Corner effects are created for the devices only at oxide-to-s-Si interfaces, leading to extreme velocities in the region having no effect on the total performance of the device. It is also perceived that in the 10 nm channel length three-fin Device (A_1_), since the lower s-Si region of the channel is not under the direct influence of the gate terminal, the electron velocity increases in this region. However, the corner effects are not effective in the upper s-Si regions. As the channel length decreases to 8 nm for Device A_8_, and the device is under the same bias as A_1_, the corner effects become more prominent across all corners of the channel; therefore, a slight increase in the leakage current and SCEs is detected. However, since tunnelling through the insulator is not observed, and the leakage is well within the limits of IRDS 2022, this behaviour is acceptable and does not degrade the output characteristics and performance of this device. Thus, it is observed that both devices A_1_ and A_8,_ though they operate beyond the limits of saturation velocities, have leakages that are within the limits as per IRDS 2022 for a 3 nm technology node and provide enriched performances, proving the novel three-fin Q-FinFET of 8 nm gate length to be superior in all aspects and to meet the standards for industrialisation.

**Figure 8:**
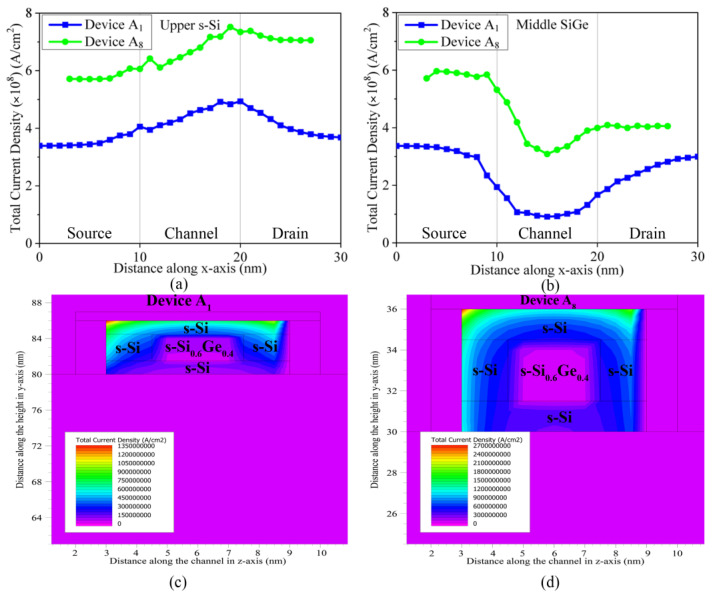
Plots of variations in total current density along the length of Device A_1_ and A_8,_ with the channel being (**a**) upper s-Si region and (**b**) middle s-SiGe; Contours of total current density across the channel of the (**c**) 10 nm three-fin FinFET and (**d**) 8 nm three-fin FinFET.

The potential of the three-fin FinFET is perceived upon integrating the electric field across the device, as presented in Figure 5d, and is observed to steadily decrease from the top of the channel to the bottom, maintaining the bulged corner effects due to the current crowding’s being minimal within the nano-channel system. The applied potential is more effective at the top and at the sides of the s-Si regions due to the TG architecture and enriched gate control, leading to better charge control and higher current densities in the device. This effect is precisely shown with the arrows in Figure 5d, indicating the process by which the decrease is effected within the active region. The potential across the channel thereby diminishes gradually and is less in the lower s-Si region, attributed to the negligible gate control, thereby decreasing the current density in this region, as is indicated in Figure 8. This layer also serves as a miniature heat sink, dissipating self-heating generated due to a Type-II hetero-system. Thus, with the sublime electrostatic-potential device performance, enrichment is increased in the novel three-fin Q-FinFET device to meet the requirements of the technological era.

The total current density of devices A_1_ and A_8_ is acquired across the length of the channel and are plotted in Figure 8a,b, while the variations along the width of the channel are shown by the contour diagrams of Figure 8c and Figure 8d, respectively. The total current density of devices A_1_ and A_8_ shows negligible diversions through the upper s-Si regions, while it demonstrates noteworthy depreciations through the middle s-SiGe regions in both devices. This leads to the creation of distinct quantum states in the novel channel system, which are clearly visible across the channel width in Figure 8c,d. The total current densities are much higher on the outer edges of the s-Si regions (under direct influence of gate control) and gradually decrease towards the middle the s-SiGe regions, which forms the distinct quantum barrier within the channel; this does not allow electron carriers to pass through. The outer s-Si regions feature high densities, and minimal current crowding-based SCEs are also detected at the s-Si/gate oxide interface corners; in comparison, the lower s-Si layer, though it suffices for electron passage, demonstrates a lower current density, as detected in Figure 8c,d. This combined effect of current density greatly increases the drive current of each fin for both the devices, suggestive of the concept of the succumbing of charge carriers due to induction of the quasi-ballistic effect, mostly in the outer s-Si regions, that provides superior gate control; thereby, the novel nano device stands as a superior alternative for the future. It is clearly noticed that the quantum states end suddenly around 0.5 nm inside the channel on the right side, decreasing the total current density in this region. This degrades the electron mobility slightly on the right side of the channel, as observed in Figure 5c, due to the quantum band’s bending in the central s-SiGe region.

According to the goals set by IRDS 2022, even with the FinFET gate lengths scaled to sub-10 nm (3 nm technology node or below), the ON currents are expected to increase with SCE-based leakages in control. Strained-silicon technology incorporated in the channel enhances the device performance, but meeting the requirement is a challenge. Thus, strained-silicon technology, together with multiple-fin structures, is employed to significantly improve the drain currents. In sub-10 nm dimensions, the inclusion of quantum effects is inescapable and therefore investigated here through analyses of the novel three-fin FinFETs with 10 nm and 8 nm channel lengths. The channel is introduced to biaxial strain by incorporating a tri-layered hetero-strained channel with dimensions of 1.5 nm-3 nm-1.5 nm layers as s-Si/s-SiGe/s-Si. The device demonstrated superior drive current enhancements with justifiable leakage currents in accordance with the 3 nm technology node proposal of IRDS 2022. The subthreshold swing and DIBL of these novel three-fin devices are also demonstrated to be on par with the single-fin Device A_9_ and IRDS 2022. The quantum investigation also resulted in higher mobility, transconductance and velocity of electrons, leading to ballistic transport through the ultra-thin s-Si layers with minimal recombination effects observed due to carrier confinement in the barrier layers of all the fins of both the devices; thereby, the devices conform to the faster switching speed and lower power in nano devices desired for the technological advancements of today. This leads to an undeniably enhanced device that stands strong to compete in the technological world of tomorrow.

## 4. Conclusions

Three-fin TG strained nano-channel Q-FinFETs are developed with 10 nm gate length (Device A_1_), while a 8 nm gate length device is also designed and further optimised for size and dimensions, considering the negligible impact of the output performance; thus, Device A_8_ is finalised. The analytical model developed validated the threshold voltage for the novel devices. Each of the three fins in the devices have identical and symmetrical structures. The devices (A_1_ and A_8_) have identical geometrical dimensions of 1.5 nm/3 nm/1.5 nm (total 6 nm thickness) of a three-layered s-Si/SiGe/s-Si channel. The fins are separated by 4 nm (1 nm SiO_2_ and 2 nm gate metal) in the devices. The ON currents are determined to be 466.63 µA/µm for Device A_1_, and 627.25 µA/µm for Device A_8_, which represents a ~154% and ~242% enhancement, respectively, over the single-fin 10 nm HOI FinFET (Device A_9_). The leakage currents of both devices A_1_ and A_8_ are determined to be 1.54 and 3.49 nA/µm, respectively, which is within the limits proposed by IRDS 2022 standards for the 3 nm technology node, thereby resulting in an efficient device. The subthreshold swings of the three-fin devices are also beneath the IRDS 2022 specified values of 82 mV/decade. The SS of Devices A_1_ and A_8_ are 71 and 76 mV/ decade, respectively, while the DIBL is calculated to be 61.8 mV/V and 91.78 mV/V for A_1_ and A_8_, respectively, demonstrating excellent control over the SCEs. The I_on_/I_off_ current ratio is 3.03 × 10^5^ and 1.8 × 10^5^ for Devices A_1_ and A_8_, respectively, and present excellent switching speeds for the devices. The lower leakage currents also contribute to lower static power dissipation of the devices, thereby decreasing the overall power consumption. A maximum transconductance, g_mmax_, enhancement of 233.33% is observed in Device A_8_ at 1.020 mS/μm, which makes the device suitable for RF applications. The analyses of electron mobility, electron velocity and the total current density in the devices elucidate the quasi-ballistic transport of charge carriers in all the fins with minimal phonon scattering greatly increasing the device performance across the devices. Thus, the three-fin hetero-strained-silicon-channelled Q-FinFETs, when downscaled to 8 nm gate lengths, keep abreast of the requirements of the 3 nm technology node according to the IRDS 2022 specifications. They are superior devices and hence are strong contenders for being the future devices equipped for RF applications of today.

## Figures and Tables

**Table 1:** Feature description of three-fin devices.

Device	Description	Substrate + BOX Height (nm)
A_1_	10 nm HOI 3-fin FinFET (2 nm gap)	80
A_2_	8 nm HOI 3-fin FinFET (2 nm gap)	80
A_3_	8 nm HOI 3-fin FinFET (2 nm gap)	70
A_4_	8 nm HOI 3-fin FinFET (2 nm gap)	60
A_5_	8 nm HOI 3-fin FinFET (2 nm gap)	50
A_6_	8 nm HOI 3-fin FinFET (2 nm gap)	40
A_7_	8 nm HOI 3-fin FinFET (4 nm gap)	30
A_8_	8 nm HOI 3-fin FinFET (2 nm gap)	30
A_9_	10 nm HOI single fin FinFET [24]	80

**Table 2:** On and leakage currents of 8 nm three-fin devices.

Device	I_on_ (μA/μm)	I_off_ (nA/μm)	I_on_/I_off_ Current Ratio (×10^5^)
A_2_	629.47	3.93	1.60
A_3_	630.35	3.84	1.63
A_4_	660.70	3.86	1.71
A_5_	638.97	3.72	1.72
A_6_	643.97	3.77	1.71
A_7_	205.64	0.78	2.65
A_8_	627.25	3.49	1.80

**Table 3:** Specifications of the three-fin TG Q-FinFETs.

Symbol	Description	Device A_1_	Device A_8_
L_D_, L_S_	Length of Drain/Source	10 nm	8 nm
L_G_	Length of Channel	10 nm	8 nm
T_ox_ (SiO_2_)	Lateral Oxide Thickness	1 nm	1 nm
W_FIN_	Thickness of Silicon Fin	6 nm	6 nm
H_FIN_	Height of Silicon Fin	6 nm	6 nm
G_FIN_	Gap Between Fins	2 nm	2 nm
N_A_	Doping of Channel	1 × 10^15^ cm^−3^	1 × 10^15^ cm^−3^
N_D_ (Source)	Doping of Source	5 × 10^18^ cm^−3^	5 × 10^18^ cm^−3^
N_D_ (Drain)	Doping of Drain	1 × 10^18^ cm^−3^	1 × 10^18^ cm^−3^

**Table 4:** Comparison of electrical characteristics.

Electrical Parameters	Kavalieros et al. [3]	Kumar et al. [22]	Bha et al. [23]	Nanda et al. [24]	A_1_ (This Work)	A_8_ (This Work)
V_th_ (V)	–	0.032	0.25	0.251	0.193	0.265
I_on_ (μA/μm)	165	106.5	166	183.13	466.63	627.25
I_off_ (nA/μm)	139	27.38	0.324	0.415	1.54	3.49
SS (mV/dec)	76	104	94	74.49	71	76
DIBL (mV/V)	89	146	–	55.83	61.8	91.78

## Data Availability

The datasets used and analysed during the current study are available from the corresponding author on reasonable request.
